# From molecular logic to therapeutic modulation: advances in Ub/UBL signalling

**DOI:** 10.1042/EBC20250062

**Published:** 2026-02-24

**Authors:** Benjamin M. Foster, Chiara Maniaci

**Affiliations:** 1 Manchester Cancer Research Centre (MCRC), Division of Cancer Sciences, School of Medical Sciences, Faculty of Biology, Medicine and Health, University of Manchester, 555 Wilmslow Road, Manchester M20 4GJ, U.K.; 2 MRC Protein Phosphorylation and Ubiquitylation Unit, Sir James Black Centre, School of Life Sciences, University of Dundee, Dundee DD1 5EH, U.K.

**Keywords:** post translational modification, therapeutics, ubiquitin signalling

## Abstract

Ubiquitin (Ub) and ubiquitin-like proteins (UBLs) are central regulators of cell signalling, with roles spanning proteasomal degradation, immune defence, DNA repair, autophagy, and the stress response. Their conserved β-grasp fold provides a remarkably versatile protein architecture that can be redeployed across diverse signalling pathways and modulated in disease contexts. Conjugation and removal through dedicated E1–E2–E3 and protease systems generate a rich regulatory code, further diversified by chain topology, hybrid architectures, and emerging non-canonical modifications. This special issue highlights recent advances in Ub and UBL biology, from specialised UBL pathways to host–pathogen interactions and non-protein conjugation events, as well as translational applications such as targeted protein degradation. Together, these reviews showcase the breadth, adaptability, and therapeutic potential of these small but powerful modifiers.

Nearly fifty years have passed since the discovery of ubiquitin (Ub), a small, highly conserved protein that reshaped our understanding of intracellular regulation [1]. What began as a system best known for marking substrates for proteasomal degradation has since expanded into an intricate signalling network influencing nearly every facet of cell biology, including the immune response, DNA repair, cell cycle control, autophagy, and programmed cell death [2–5]. Central to this versatility is the β-grasp fold, a compact and modular structural solution that nature repeatedly repurposes across the proteome, giving rise to the family of Ub-like (UBL) proteins [6–9]. Members, such as small ubiquitin-like modifier (SUMO), ISG15, ubiquitin-like modifier 1 (UFM1), ATG8, ATG12, URM1, FUBI, NEDD8, and FAT10, expand the functional reach of Ub-like signalling and diversify how cells regulate protein function in response to environmental and intracellular cues [10,11]. This conserved architecture has this way enabled the evolution of sophisticated enzymatic cascades, a diverse ‘modification code’ [12–14], and an exceptional degree of regulatory adaptability that can be remodelled in disease contexts and exploited therapeutically.

Whether attached transiently, incorporated into polymeric chains, or recognised through specific interaction motifs, this fold provides a modular platform for encoding, transmitting, and interpreting diverse cellular signals. This themed issue of Essays in Biochemistry highlights how the field continues to redefine the functional potential of the Ub and UBL system. The collection spans biochemical mechanisms, structural insights, pathogen exploitation, and translational innovation—together illustrating how far the field has progressed and how many conceptual frontiers remain open.

Several contributions examine SUMO biology, one of the most extensively studied UBL pathways, from complementary angles, revealing its multifaceted regulatory logic [15]. Two complementary reviews by Cissé, Mishra, and Suskiewicz provide a comprehensive overview of the SUMO system from different vantage points [16,17]. Their first article focuses on core non-covalent interactions between SUMO and its (de)conjugation machinery, detailing how E1, E2, and SENP proteases position SUMO for activation, transfer, and cleavage [17]. The accompanying review extends this framework to SUMO-interacting motifs (SIMs) and emerging SIM-independent interfaces that diversify SUMO signalling outputs [16]. Together, these pieces illustrate how a small modifier can exert wide-ranging effects through finely tuned binding modules and regulated assembly of multi-SUMO architectures.

This mechanistic foundation is further expanded in the review by Bhachoo and Garvin, who explore the roles of SUMO in DNA double-strand break repair (DSB), a striking illustration of regulatory complexity in genome maintenance [18]. They discuss how waves of SUMOylation coordinate factor recruitment, stabilise repair complexes, and regulate the amplitude of the DSB-SUMOylation response. Their analysis showcases the SUMO system as a paradigm of how a UBL can choreograph complex, multistep decision-making in genome stability pathways.

The issue further expands into UBLs with specialised cellular roles. Kwasna, Ravichandran, Biela, and Glatt provide a structural and mechanistic tour of the ancient Urm1–Uba4 system, a hybrid that blends features of prokaryotic sulphur-carrier proteins with eukaryotic UBL chemistry [19,20]. Their review illustrates how Urm1’s unique activation cycle enables both tRNA thiolation and oxidative-stress-linked protein URMylation, expanding the conceptual boundaries of what UBLs can do. Some UBLs, such as UFM1, have highly specific cellular roles tight to specific cellular compartments or organelles, enabling very localised signalling [21–23]. Komatsu and Mao explore this in the context of UFM1 and its role at the endoplasmic reticulum, where it plays a key role in modulating protein synthesis, folding, and degradation signalling [24].

While predominantly a protein PTM, there is increasing evidence for non-canonical modifications by Ub/UBLs, such as non-lysine protein modifications and non-protein ubiquitylation [25–28]. Zhu, Chatrin, Smith, and Ahel discuss how ubiquitylation intersects with ADP-ribosylation, including the formation of hybrid ADPr-Ub modifications [29]. These dual signals, catalysed by Deltex ligases in cooperation with PARPs, exemplify how Ub’s chemistry is adapted to create composite regulatory outputs. In addition, the identification of Ub/UBL enzymes from other clades of life, e.g. SARS-CoV PLPro [30,31], has highlighted how pathogens may hijack the cellular machinery to enable infection and has also provided useful tools for the detection and monitoring of Ub/UBL signalling in cells [32–34]. Bhark, Lacoursiere, and Pruneda review how bacterial effectors manipulate the Ub and UBL code—targeting conjugation cascades, altering modifier surfaces, or hijacking host ligases. Their survey underscores how studying pathogen strategies continues to illuminate fundamental principles of Ub/UBL regulation.

An exciting avenue that has been utilised in ‘drugging the undruggable’ is the development of heterobifunctional molecules and molecular glues that enable targeting of proteins for rapid degradation via the proteasome [35–37]. PROTACs (proteasome targeting chimeras) predominantly target proteins to the E3 ligases CRBN or VHL, enabling rapid Ub chain formation and subsequent proteasome-mediated degradation, while recycling the PROTAC molecule. This exciting field of research, both in academic contexts and in related spinouts and pharmaceutical companies, has paved the way for potential translational and clinical applications of hijacking Ub/UBL signalling. Kozicka charts the evolution of molecular glue degraders from serendipitous discoveries to more systematic design principles, revealing how small molecules can stabilise protein interfaces to redirect ligase activity. Complementarily, Liu explores degron mimetics, outlining how endogenous degrons and ligase recognition motifs can inspire targeted degradation strategies that reshape protein fate with precision [38].

Overall, from canonical conjugation pathways to hybrid modifications and from bacterial virulence mechanisms to engineered degraders, the contributions in this issue underscore a unifying principle: the β-grasp fold is a versatile structural currency that cells, pathogens, and chemists alike can repurpose to encode regulatory information and modulate protein fate. While this group of mini reviews gives a small snapshot of some of these and the mechanisms behind them, it also illustrates how understanding how these modifiers are written, edited, and interpreted not only illuminates fundamental cell biology but also provides a blueprint for rewiring signalling for therapeutic benefit. From structural insights to chemical innovation, the Ub/UBL field continues to expand the vocabulary of cellular regulation and the opportunities for translating it into medicine.

## Open Access

Open access for this article was enabled by the participation of the University of Manchester and the University of Dundee in an all-inclusive *Read & Publish* agreement with Portland Press and the Biochemical Society under a transformative agreement with JISC.

## Abbreviations

ATG8/12: autophagy-related protein 8/12
ATP: adenosine triphosphate
DSB: double-strand break repair
FAT10: human leukocyte antigen F locus adjacent transcription 10
FUBI: Finkel-Biskis-Reilly murine sarcoma virus (FBR-MuSV) ubiquitously expressed
ISG15: interferon-stimulated gene 15
NEDD8: neural precursor cell expressed and developmentally down-regulated 8
PARP: poly ADP-ribose polymerase
PROTAC: proteasome-targeting chimera
PTM: post-translational modification
SUMO: small ubiquitin-like modifier
Ub: ubiquitin
UBL: ubiquitin-like protein
UFM1: ubiquitin-like modifier 1
URM1: ubiquitin-related modifier 1
